# P-336. Acceptability and Usability of a Latino MSM-tailored HIV Prevention App: A Pilot Study

**DOI:** 10.1093/ofid/ofaf695.555

**Published:** 2026-01-11

**Authors:** Valeria D D Cantos, Humberto Posada-Orozco, Isabella Batina, Natalie Sanchez Farez, Eric Rangel, Patrick S Sullivan, Andres Camacho-Gonzalez, Aaron J Siegler

**Affiliations:** Emory University School of Medicine, Atlanta, Georgia; Emory University School of Medicine, Atlanta, Georgia; Emory University School of Medicine, Atlanta, Georgia; Duke University School of Medicine, Durham, North Carolina; Latino Linq, Atlanta, Georgia; Emory University Rollins School of Public Health, Atlanta, GA, USA., Atlanta, GA; Emory University School of Medicine, Atlanta, Georgia; Emory University, Atlanta, Georgia

## Abstract

**Background:**

Human Immunodeficiency Virus (HIV) incidence is increasing among Latino gay, bisexual and other men who have sex with men (LMSM) in the Atlanta metropolitan area. Mobile phone applications represent an innovative tool to promote pre-exposure prophylaxis (PrEP), HIV testing, and condom use. The objective of the study was to assess the acceptability and usability of Saludfindr, an Android-based HIV prevention app tailored to the needs of LMSM in the Atlanta area.

**Methods:**

We recruited adult LMSM to interact with the app for 4 months. Saludfindr included initial and periodic health assessments, provision of suggestions regarding PrEP, HIV testing, and condom use, in-app product ordering, customized motivational messages, a customized sexual health clinic list, and a “Contact Us” button. To assess acceptability, we measured usage of each app feature, PrEP and HIV testing uptake, and participant ratings of the app’s usefulness. We assessed usability with the System Usability Score (SUS).

**Results:**

We enrolled 31 participants; median age was 27 years [interquartile range (IQR) 24.5-32], 97% were cisgender men, 81% (25/31) identified as MSM, and 61% (19/31) used the app in Spanish. All participants completed the initial health screening, with 84% (26/31) and 77% (24/31) completing the 2- and 4-month health screenings, respectively. Of all participants, 52% (16/31) and 23% (7/31) ordered condoms and home HIV tests through the app at least once, respectively. During the study period, 71% (22/31) of participants got tested for HIV, of whom 68% (15/22) accessed it through clinic-based HIV testing. Of the participants not on PrEP at baseline, 41% (7/17) initiated PrEP during the study, and all of them did so at one of the clinics listed in the app. Saludfindr reached a SUS score of 74/100 (excellent).

**Conclusion:**

Saludfindr was highly acceptable and usable among LMSM participants in the Atlanta area. In-app assistance to access PrEP and clinic-based HIV testing referrals were well received. Further efforts are needed to increase users’ self-efficacy with home HIV self-testing.

**Disclosures:**

Valeria D. D. Cantos, MD, FIDSA, Gilead Sciences Inc.: Grant/Research Support Patrick S. Sullivan, DVM, PhD, Gilead Sciences Inc.: Grant/Research Support|Gilead Sciences Inc.: Speaker fees|Merck: Grant/Research Support|ViiV Healthcare: Grant/Research Support Andres Camacho-Gonzalez, MD, MSc, Gilead Sciences Inc.: Grant/Research Support|GSK: Grant/Research Support|Merck: Grant/Research Support|ViiV: Grant/Research Support

